# Quantitative and qualitative electroencephalography in the diagnosis and monitoring of depression. A modern approach to clinical neurophysiology

**DOI:** 10.3389/fnhum.2025.1624434

**Published:** 2025-08-08

**Authors:** Marta Kopańska, Danuta Ochojska, Izabela Sarzyńska, Oliwia Bartkowska, Jacek Szczygielski

**Affiliations:** ^1^Faculty of Medicine, Department of Medical Psychology, Collegium Medicum, University of Rzeszów, Rzeszów, Poland; ^2^Laboratory of Neurophysiology, Clinimetrics and Microsurgical Training, Centre for Innovative Research in Medical and Natural Sciences, Collegium Medicum, University of Rzeszów, Rzeszów, Poland; ^3^Faculty of Health Sciences and Psychology, Collegium Medicum, University of Rzeszów, Rzeszów, Poland; ^4^Student Research Club “Reh-Tech”, Collegium Medicum, University of Rzeszów, Rzeszów, Poland; ^5^Faculty of Medicine, Department of Neurosurgery, Collegium Medicum, University of Rzeszów, Rzeszów, Poland; ^6^Faculty of Medicine and Saarland University Medical Centre, Department of Neurosurgery, Saarland University, Homburg, Germany

**Keywords:** depressive disorders, EEG, QEEG, neurophysiological biomarkers, frontal alpha asymmetry, brain wave activity, diagnosis of depression

## Abstract

**Context:**

Depressive disorders are one of the greatest public health challenges, affecting more than 300 million people worldwide. Traditional diagnostic methods are based on subjective clinical assessments, which limits their accuracy and reproducibility. Therefore, there is an urgent need to implement objective, easily accessible diagnostic tools. One such tool is quantitative electroencephalography (QEEG), which allows for the analysis of the bioelectrical activity of the brain in a non-invasive and precise manner.

**Methods:**

In this narrative review, the latest research on the use of QEEG and traditional EEG in the assessment of patients with major depressive disorder (MDD) was analyzed. The literature search was carried out in the PubMed and SpringerLink databases, focusing on articles investigating the correlations between spectral EEG properties (alpha, beta, theta, delta, gamma waves) and symptoms of depression.

**Results:**

The literature review indicates the presence of characteristic patterns of brain activity in patients with MDD, such as alpha wave asymmetry in the frontal areas, increased beta band activity, and changes in the theta and delta waves. This indicates the potential use of these parameters as biomarkers for early detection and monitoring of therapy effectiveness.

**Conclusions:**

QEEG and classical EEG may play an important role in the diagnosis and treatment of depression, supporting the development of personalized therapeutic strategies. Despite promising results, further research on the standardization of methods and validation of neurophysiological indicators is needed to enable their wider application in psychiatric clinical practice.

## 1 Introduction

One of the main challenges facing modern psychiatry today is the identification of biomarkers that will enable the accurate diagnosis of major depressive disorder (MDD). The diagnostic methods currently used are based on subjective assessments, which limits their reliability and leads to an incomplete reflection of the patient's real condition (Boby and Veerasingam, 2025).

Depression is currently one of the most commonly diagnosed mental disorders in the world, affecting people of all ages. According to the World Health Organization (WHO), more than 300 million people suffer from depression. MDD is a serious challenge, both socially and economically. It is a disease that not only leads to a reduced quality of life, but also generates significant economic costs, including reduced productivity, absenteeism and increased healthcare expenditure. It mainly manifests itself in general discouragement, a feeling of uselessness, a pessimistic vision of the future, a decrease in concentration, and a constant feeling of guilt. Patients also experience physical symptoms such as pain, fatigue, and sleep problems (de Aguiar Neto and Rosa, 2019; Rakel, 1999). Patients with MDD often suffer from cognitive impairments, which include not only a reduced ability to think, but also a weakened ability to make decisions, which may result from the fact that patients mainly focus their attention on negative emotions. Therefore, people suffering from MDD are characterized by a specific way of thinking, based on persistent and intense consideration of the causes and consequences of their negative emotions (Dehn and Beblo, 2019).

According to the World Health Organization around 20 percent of people with MDD die, while around 35 percent attempt suicide every year (Boby and Veerasingam, 2025). It is therefore important that depressive disorders are diagnosed effectively and quickly, as this makes it possible to prevent serious health consequences associated with the course of the disease. Depression is characterized by moderate and severe episodes and is often treated with pharmacological agents or psychosomatic therapies. For many people, it can become a chronic condition or last a lifetime, often with relapses. On average, people suffering from depression experience four to five depressive episodes in their lifetime (Ghiasi et al., 2021). MDD is classified into three main categories: severe, moderate and mild. Patients with severe depressive disorders can be diagnosed relatively easily and quickly. However, identifying moderate and mild episodes of depression is much more difficult, which is why it is important to develop tests that enable quick and effective detection of depression (Wu et al., 2022).

The causes of depression are complex and multifactorial, and the literature on the subject is not clearly systematized, but it is generally known that its development can be influenced by various factors, such as genetics, long-term stress, trauma or adverse environmental conditions (Remes et al., 2021). It is believed that one of the factors of depression is the ongoing active inflammatory processes in the body, which weaken the blood-brain barrier (Anderson, 2016; Bobińska et al., 2016).

Other studies have shown that there is a relationship between chronic stress and depressive disorders (Hansson et al., 2015). Interestingly, it is believed that the brains of people suffering from depression are characterized by impaired signal transmission between neurons, which may result from the malfunctioning of neurotransmitters responsible for mood regulation, pleasure sensation, reward system and executive functions (Durisko et al., 2015). People suffering from depression experience elevated levels of cortisol at night. This contributes to structural changes in brain areas responsible for emotional processing, such as the enlargement of the amygdala in the anterior part of the temporal lobes (Yasin et al., 2023).

According to the Diagnostic and Statistical Manual of Mental Disorders, 5th Edition (DSM-5) criteria, depression is diagnosed when five or more symptoms of MDD occur for about 2 weeks, including depressed mood or loss of interest, causing significant suffering or impairment of functioning, not resulting from another cause (Kendler, 2016). Depression is now considered a risk factor for cardiovascular diseases such as hypertension, arteriosclerosis and myocardial infarction. Depression can also weaken the immune system, increasing susceptibility to infections and chronic inflammation. It is also closely related to metabolic disorders such as type 2 diabetes, as well as neurological problems. In addition, depression often coexists with anxiety disorders, insomnia and chronic pain syndrome, which significantly worsens the quality of life of patients (Carney, 2017; Ma and Li, 2017; McGregor et al., 2014; Levenstein et al., 2001; Berntson et al., 2017; Anderson et al., 2001). That is why it is so important to diagnose depression quickly, which will allow for the implementation of appropriate treatment at an earlier stage, minimizing the negative consequences for the patient and society.

The QEEG seems to be a helpful tool in the diagnosis of the neurological mechanisms causing depression. Many scientists are using and QEEG to find biomarkers that may play a key role in the diagnosis and treatment of depressive disorders in the future. It is now known that depression affects three areas of the brain: the prefrontal cortex at the front of the frontal lobe of the frontal bone, the amygdala at the front of the temporal lobe and the hippocampus in the temporal lobe (Wu et al., 2022).

**Figure 1:**
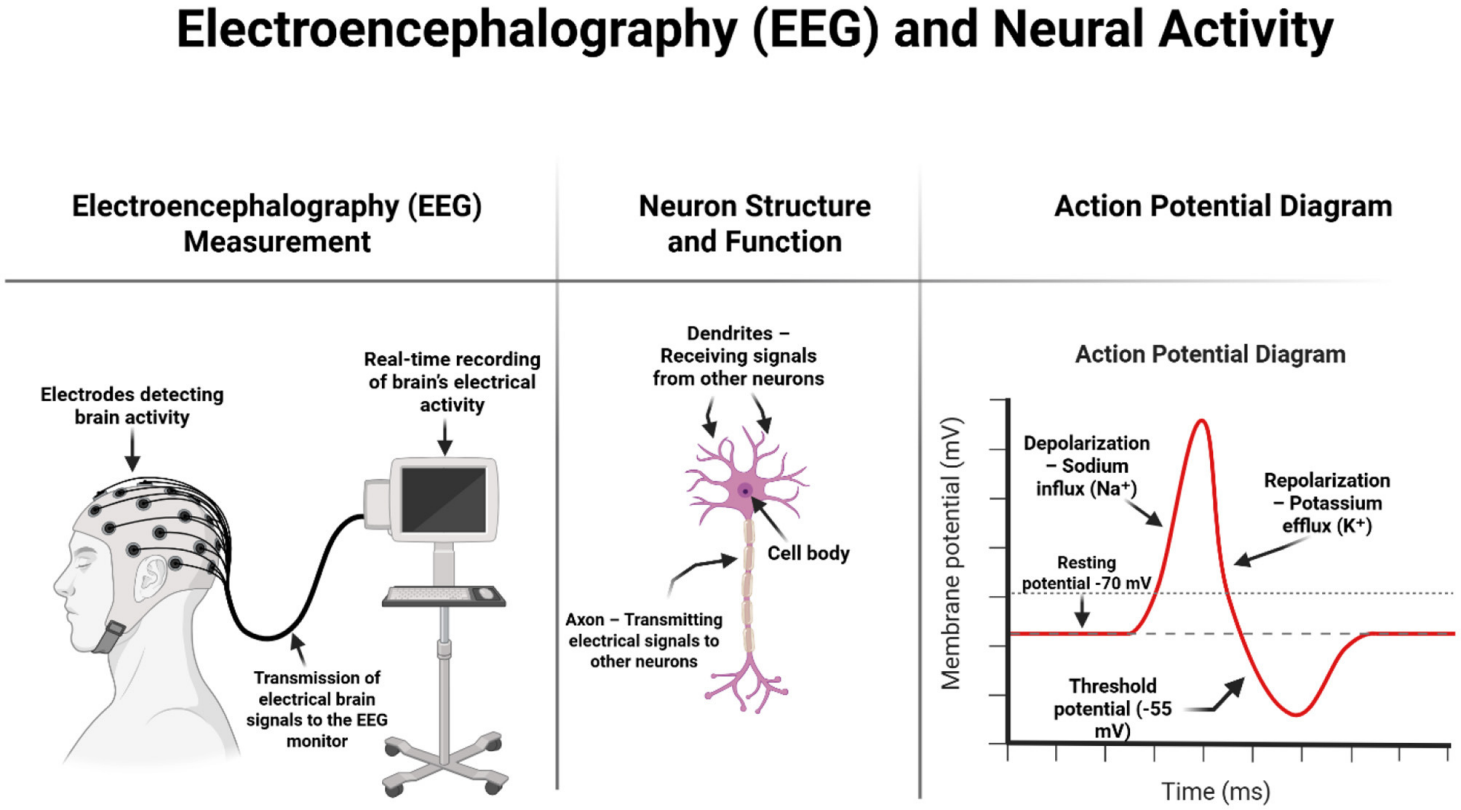
Schematic representation of EEG measurement, neuron structure, and the action potential process. On the left, the diagram shows how surface electrodes placed on the scalp detect the brain's electrical activity, which is transmitted in real time to an EEG recording system. The center illustrates the basic structure of a neuron, including dendrites (which receive input), the cell body (soma), and the axon (which transmits signals). On the right, a graph of the action potential explains key phases of neuronal firing: resting state, depolarization due to sodium influx, and repolarization due to potassium efflux. This electrophysiological process is the basis of the brain signals captured during EEG recording. Image created using Biorender.

Traditional electroencephalography involves monitoring brain activity in different areas of the skull and recording the currents generated by brain neurons using electrodes placed on the surface of the head, which are then amplified by an electroencephalograph. Traditional EEG is a widely available, easy-to-use technique (Figure 1) (Fingelkurts and Fingelkurts, 2022). In contrast, quantitative electroencephalography is a test that can assess the functioning of the central nervous system. QEEG can be used to assess brain activity and examine the correlations between areas of the cerebral cortex. QEEG is also known as “mapping” of the bioelectrical activity of the brain. It refers to the numerical analysis and visual transformation of raw electroencephalographic signals. An additional advantage of this test is that it is non-invasive (Kopańska et al., 2023). In QEEG analysis, the most commonly used measures are spectral power, which reflects the intensity of activity within a given frequency band, and coherence, which indicates the phase consistency between signals recorded at different points on the scalp (Leuchter et al., 2012).

Several researchers point out that various impairments in brain performance and emotional state are noticeable in the bioelectric activity of the brain. Therefore, both EEG and QEEG are valuable tools for obtaining information on changes in neuronal functioning in the presence of MDD (Bachmann et al., 2017). As early as the 1950s, some authors were already considering the use of electroencephalography in the diagnosis of MDD (Williams, 1954). Nowadays, not only plain EEG is used for diagnostic workup of depressive disorders. Meanwhile modified version of EEG, namely quantitative electroencephalography was included in the list of diagnostic tools for MDD assessment (Deslandes et al., 2004; Leuchter et al., 2009b; Widge et al., 2019).

Both of these tests play an important role in the precise identification of optimal therapeutic strategies, which contributes to more targeted and effective treatment (Schiller, 2019).

The aim of this review is to summarize the latest research on the application of traditional EEG and QEEG in the diagnosis of depression and to identify specific biomarkers characteristic of MDD. Currently, there is a growing interest in objective methods for assessing brain function in MDD, and this article seeks to organize the available knowledge and indicate directions for further research.

## 2 Materials and methods

This systematic review was conducted in accordance with the PRISMA (Preferred Reporting Items for Systematic Reviews and Meta-Analyses) guidelines and the recommendations of the Cochrane Collaboration Handbook for Systematic Reviews of Interventions. The literature search included an analysis of scientific articles on the use of electroencephalography and quantitative electroencephalography in the diagnosis and monitoring of depression.

**Figure 2:**
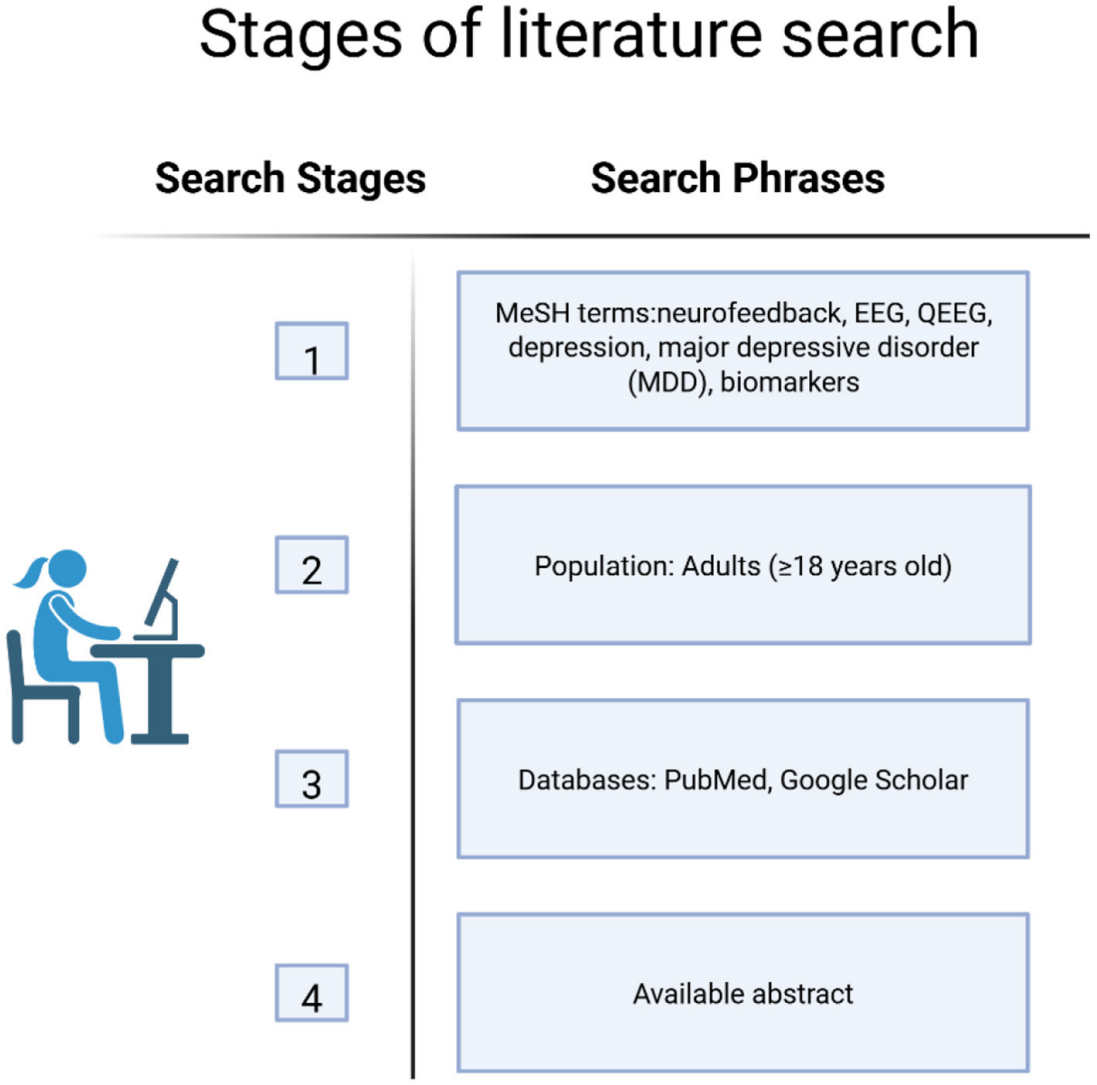
The process of identifying relevant scientific literature in PubMed and Google Scholar databases. Search terms were selected based on MeSH terminology and inclusion criteria related to QEEG, EEG and biomarkers of MDD. Image created using Biorender.

The literature search was conducted in two key databases: PubMed and SpringerLink, to ensure broad access to peer-reviewed publications of high scientific value. In order to identify relevant studies, various combinations of keywords were used, taking into account both general concepts related to traditional EEG and depression, as well as more specific aspects concerning neurophysiological biomarkers. Examples of search phrases used included: “EEG/QEEG depression”, “EEG biomarkers depression”, “QEEG MDD”, “frontal alpha asymmetry depression”, “EEG spectral analysis in major depressive disorder”, “brain oscillations in mood disorders” (Figure 2).

The review included studies that met the following criteria: original studies that analyzed EEG or QEEG activity in adults (≥18 years old) with depression, peer-reviewed publications available in English or Polish, works analyzing specific brainwave bands (alpha, beta, theta, delta, gamma) and their potential diagnostic significance, studies that included traditional EEG spectral analysis methods, including frontal asymmetry and brainwave synchronization indices. Studies with a large study group were also considered important, enabling comparison of results between different clinical subgroups, which could increase the scientific value of the conclusions. However, the following studies were not included in the review: studies conducted on children and adolescents (< 18 years of age), due to differences in neurophysiological development, without access to the full text or publications that were not scientifically reviewed, conference papers, case studies and narrative reviews (with the exception of meta-analyses).

A total of 4296 studies were identified in the databases (PubMed: 2583, SpringerLink: 1713). After removing 1015 duplicates, 3281 studies remained for further analysis. Subsequently, 495 studies were rejected due to lack of access to the full text and 420 studies were excluded because they involved an unsuitable population. Ultimately, 2366 studies were screened for eligibility. During the initial selection (review of titles and abstracts), 1500 studies were excluded due to lack of relevance to the study topic. The full texts of the remaining 866 studies were subjected to a detailed evaluation. After the full-text evaluation, 696 studies were rejected because they did not meet the inclusion criteria:

- Studies conducted on children and adolescents (*n* = 400)
- Lack of complete traditional EEG data (*n* = 200)
- Low methodological quality (*n* = 96)

**Figure 3:**
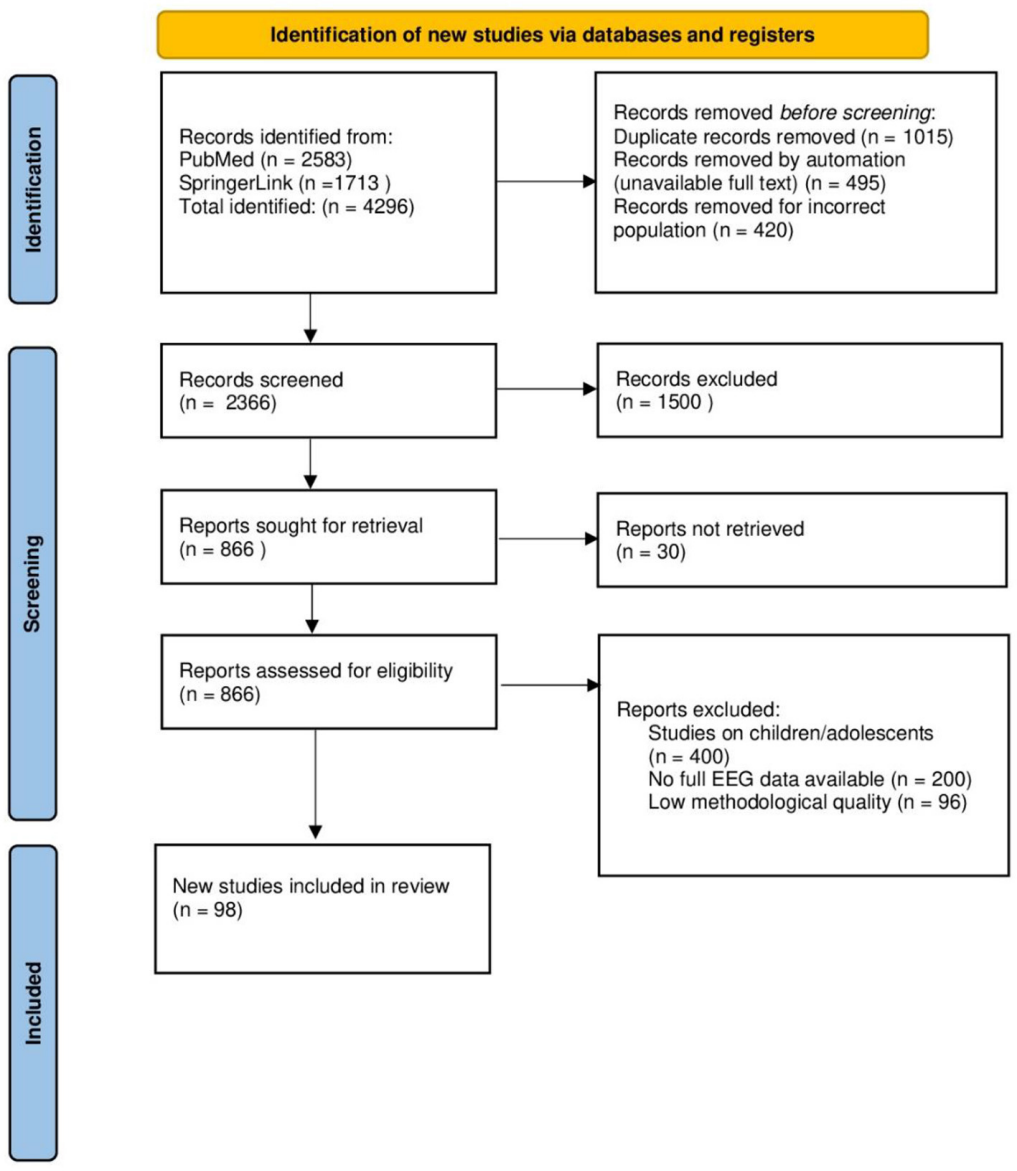
PRISMA chart for included studies.

Ultimately, according to the inclusion and exclusion criteria, 98 studies were included in the systematic review, of which 69 were subjected to detailed analysis, and 29 were used as contextual sources in the introduction (Figure 3).

## 3 Results

The aim of this paper is to analyse the results of studies that evaluated the usefulness of EEG and QEEG as diagnostic tools in the diagnosis of depressive disorders. The focus was on identifying specific patterns of bioelectrical brain activity characteristic of MDD. The results were categorized according to activity in individual brainwave bands, taking into account their potential diagnostic and prognostic significance.

Quantitative electroencephalography is becoming an increasingly common tool in psychiatric neurodiagnostics. The literature emphasizes its usefulness in assessing the current state of brain function and the possibility of early detection of mood disorders (Kopańska et al., 2023).

### 3.1 Frequency domain analysis

One of the most commonly used methods in QEEG is frequency domain analysis, which is based on the fast Fourier transform. It allows for the assessment of EEG signal power within specific frequency bands, which reflect the activity of various neural circuits, particularly thalamocortical and cortical networks (Smailovic and Jelic, 2019).

Studies conducted over the past 10 years using traditional electroencephalography in patients with depression indicate significant methodological issues that hinder the comparability of results. The main limitations include the diversity of diagnostic criteria within study groups, the lack of appropriate control groups, and substantial variability in EEG techniques used across studies (Thoduparambil et al., 2020; Pollock, 1990). Despite these limitations, increasing attention is being given to the identification of specific biomarkers that could aid in earlier diagnosis.

Soares de Aguiar Neto and Garcia Rosa emphasize that alpha waves in EEG recordings have significant diagnostic relevance in the context of depressive disorders (de Aguiar Neto and Rosa, 2019). Alpha waves occur in the frequency range of 8–12 Hz and are observed with eyes closed, in a state of relaxation and wakefulness. They most commonly appear in the occipital region. In individuals suffering from depression, researchers observe alpha wave asymmetry in the frontal part of the brain (van der Vinne et al., 2019). Kana et al. identified differences in alpha waves between a group of healthy individuals and a group of individuals with depression. It was found that alpha waves in the MDD group were lower compared to the control group. Additionally, these abnormalities were observed in the frontal, parietal, occipital, and temporal regions (Kan and Lee, 2015). An international team of researchers specializing in neurobiology and psychiatry, while analyzing the role of alpha waves in major neuropsychiatric disorders, recorded reduced activity of these waves in depression, particularly in the prefrontal cortex. These findings suggest that such a wave pattern may lead to difficulties in emotion regulation and impaired cognitive functioning (Ippolito et al., 2022). According to some studies, qEEG can be used to predict the effectiveness of pharmacological treatment in MDD. Female patients who do not respond to treatment are characterized by greater alpha wave power in the left frontal lobe compared to the right (Ip et al., 2021). Similarly, in the study by Arikan et al., it was shown that patients with MDD who respond to treatment exhibit significant changes in cortical activity in the theta, alpha, and high beta bands, with qEEG improvement ranging from 15 to 67% (Arikan et al., 2025). In turn, treatment with citalopram, a selective serotonin reuptake inhibitor (SSRI), results in an increase in alpha power (Pariante et al., 2012).

In a study involving 1,008 patients with MDD and 336 individuals in the control group, traditional EEG was performed, and it was observed that patients with MDD had elevated theta values in the frontal cortex. The authors concluded that increased theta waves in the frontal region were associated with a lack of response to treatment. On the other hand, other researchers noted that low theta wave values in the frontal cortex occur in individuals who respond poorly to antidepressant therapy (Arns et al., 2015). Theta waves fall within the frequency range of 4–8 Hz and are most commonly recorded during drowsiness, relaxation, or meditation (Kopańska et al., 2023). In MDD, a significant increase in spectral power in the theta frequency bands can be observed in the parietal and occipital regions, both with eyes open and closed (Grin-Yatsenko et al., 2009). Recent studies suggest a correlation between depression and pathological theta wave values. In their study, Melinda L. Morgan et al. examined 104 individuals suffering from depression using quantitative electroencephalography. The obtained results were compared with a control group. The authors noted that theta wave correlation in the prefrontal regions may serve as a biomarker of depression, as it is associated with the pathophysiology of the disorder and with predicting treatment response. Interestingly, absolute prefrontal power was higher in women than in men (Morgan et al., 2005).

Suzuki et al. conducted a quantitative EEG analysis on three groups of participants: 27 patients with melancholic depression, 21 patients in remission, and 17 individuals without mental disorders. The latter two groups served as the control group. After completing the study, the authors observed that individuals suffering from depression were characterized by higher theta power in the bilateral frontal regions compared to the remission group. Moreover, they noted that individuals who had experienced depression showed lower theta and alpha power in the bilateral frontal regions and lower alpha-1 power in the parietal regions compared to the control group, which may indicate specific neural dysfunctions related to emotional regulation and information processing in this population (Suzuki et al., 1996). Other authors also point out that patients with MDD are characterized by anomalies in the theta frequency range. In patients with depression, lower theta activity and faster activity in the frontal areas of both hemispheres and the posterior part of the right hemisphere can be observed compared to the control group (Begić et al., 2011; Ohashi, 1994; Yamada et al., 1995; Fingelkurts et al., 2007). Interestingly, patients with MDD show elevated theta wave values in frontal regions (Suzuki et al., 1996).

Begić et al. observed increased beta wave activity in patients with depression (Begić et al., 2011). Therefore, Cai H. et al. conducted an EEG study in patients with MDD, focusing, among other things, on beta wave analysis. The authors noted that beta wave activity, which is associated with mental activity and focus, may have atypical patterns in patients with depression, indicating disturbances in cognitive activity (Cai et al., 2018). In the study of Cai et al., the concept of simplified three channel EEG collection covering frontal area has been implemented. This is in concordance with canonical prefrontal EEG asymmetry pattern as the hallmark of affective disorders, including depression (Davidson, 1998). The anatomical attribution of beta-wave relevant changes may be however more complex, including not only frontal, but also temporoparietal sites, including their bilateral interconnectivity (Li et al., 2017).

An intriguing finding is that Internet addiction combined with depression is associated with specific brainwave patterns. Studies have shown that these patients exhibit increased relative theta power and decreased alpha power across all brain regions (Lee et al., 2014). As early as the end of the 20th century, researchers noticed a connection between the occurring traditional EEG abnormalities in the delta wave range in people suffering from depression. Kwon et al. showed that delta wave activity is increased in people with MDD and that the dominance of these waves is more common in the right hemisphere (Kwon et al., 1996). Depression patients are characterized by a higher delta and theta spectral power in the left superior temporal gyrus at the level of Brodmann areas. Increased activity of these waves may reflect difficulties in concentration, reduced ability to process emotional stimuli and problems with mood regulation (Spironelli et al., 2021). In contrast, a study of 60 menopausal patients diagnosed with depression observed anomalies in EEG activity. These included an increase in the relative values of delta/theta and beta waves, a decrease in alpha band activity, and a slowing of the center of gravity of delta/theta wave activity (Saletu et al., 2010). An interesting example of EEG research is the analysis of the effect of music therapy on functional brain connectivity in patients with MDD. In a study involving 8 patients with depression and 8 control subjects, a greater delay in the delta wave phase was observed in the patients with depression, which may indicate characteristic changes in brain activity in this group (Simmatis et al., 2023). It is worth noting that lower delta wave activity has also been identified as a potential indicator of susceptibility to depressive episodes in people suffering from this disease (Buysse et al., 1997). An EEG study conducted during sleep in hospitalized patients diagnosed with depression who were not taking medication showed that both low and high delta sleep index values are characteristic of people suffering from depression. This could potentially be a useful diagnostic marker in identifying this disorder (Nissen et al., 2001).

According to neurobiological research, a reduced level of gamma waves is associated with depression, and people suffering from this disorder often show poorer concentration than healthy people. Therefore, gamma waves can significantly affect the effectiveness of classification processes (Jiang et al., 2021). Gamma brain wave activity will help diagnose depression more than other frequency bands (Malik et al., 2018). Researchers at the University of Wisconsin-Madison examined 17 patients using a 128-channel EEG. After conducting the study, they noticed that people suffering from depression are characterized by a significantly reduced gamma frequency density (Pizzagalli et al., 2006). Numerous studies indicate that gamma rhythms in people with unipolar depression differ from those observed in bipolar disorder, including episodes of bipolar depression (Isomura et al., 2016; Liu et al., 2012; Lee et al., 2010). Listening to an instrumental piece of music by 19 people with diagnosed unipolar depression caused an increase in gamma wave activity in the prefrontal area. This result suggests that such an intervention can modulate cortical activity, which potentially indicates the therapeutic effect of music on brain function in depression (Mosabbir et al., 2022). The analysis of gamma and delta bands shows increased gamma activity in patients with MDD, especially in response to emotional stimuli. Other authors, on the other hand, observed a reduced resting gamma density in people with depression, which may indicate a reduced activity of tonic processes in the anterior regions of the cingulate gyrus (Akdemir Akar et al., 2015). Individuals with MDD exhibited significantly increased current density in the delta, theta, alpha, beta1, and beta2 frequency bands compared to control subjects in the anterior cingulate cortex and prefrontal cortex (Korb et al., 2008). Roemer et al., on the other hand, used traditional EEG to examine elderly people diagnosed with depression. The patients were observed to have lower delta values and higher theta wave values, which has also been noted by other researchers, as shown in Table 1 (Roemer et al., 1992).

**Table 1:** Summary of EEG frequency domain analysis findings in major depressive disorder.

**Researchers**	**Aim**	**Material and methods**	**Results**
de Aguiar Neto and Rosa (2019)	Review of the use of EEG biomarkers in depression.	Review of EEG literature on depression.	EEG biomarkers, especially those related to alpha waves, correlate with depression
van der Vinne et al. (2019)	Investigating frontal alpha asymmetry in patients with depression during antidepressant treatment	Traditional EEG analysis before and after treatment of patients with depression	Frontal alpha asymmetry may be a useful indicator of the effectiveness of depression treatment
Kan and Lee (2015)	Investigating alpha wave decline in depression using traditional EEG	EEG analysis focused on alpha wave frequencies	Reduced alpha wave activity may be a potential indicator of depression
Ippolito et al. (2022)	Review of the role of alpha oscillations in neuropsychiatric disorders	Review of research from the last 10 years	Alpha oscillations play a key role in the occurrence of MDD
Ip et al. (2021)	To evaluate the prognostic value of previously suggested QEEG markers in predicting antidepressant response in patients with MDD.	Non-randomized, open-label prospective clinical study with 100 MDD patients; 79 included in per-protocol analysis. EEG was recorded before treatment.	Frontal alpha asymmetry appears to be a promising biomarker in women with MDD. Greater pre-treatment alpha power in the right vs. left frontal lobe was linked to better clinical outcome at 8 weeks.
Arikan et al. (2025)	To compare qEEG data from MDD patients and healthy controls before and after treatment, assessing how treatment response affects neuronal activity.	72 patients aged 18–60, medication-free for ≥2 weeks. qEEG was recorded pre- and post-treatment.	Responders showed significant cortical activity changes (theta, alpha, high beta) toward patterns seen in healthy controls (improvement range: 15–67%). Non-responders showed minimal changes (range: 38–46%).
Pariante et al. (2012)	To examine whether citalopram treatment affects glucocorticoid receptor (GR) activity in the brain by analyzing EEG	20 healthy men received citalopram or placebo for 4 days, followed by cortisol. Traditional EEG and working memory were assessed.	Citalopram reduced the increase in alpha power
Arns et al. (2015)	Investigation of frontal theta and anterior cingulate activity in depression.	Theta EEG analysis	Theta in frontal areas had prognostic significance for depression
Grin-Yatsenko et al. (2009)	Early detection of depression using traditional EEG	EEG analysis in patients with early depression	In parietal and occipital areas, with eyes open and closed, a significant increase in the power of the spectrum in the theta frequency bands can be seen
Morgan et al. (2005)	Investigation of the influence of age, gender and depression on quantitative EEG	Traditional EEG analysis taking into account demographic factors	The correlation of theta waves in the prefrontal areas can act as a biomarker for depression
Suzuki et al. (1996)	Comparison of traditional EEG characteristics in the depression and remission phase in patients with depression	Quantitative EEG in patients with different conditions	Depressed individuals are characterized by higher theta power in bilateral frontal areas
Begić et al. (2011)	Comparison of QEEG in schizophrenia and depression	QEEG analysis in patients with different diagnoses	Depressed patients show increased delta and theta activity
Ohashi (1994)	Comparison of traditional EEG activity in first-degree relatives of patients	Resting EEG analysis in different groups	Depressed patients show reduced theta activity
Yamada et al. (1995)	EEG analysis in patients with pre- and post-menopausal depression.	Characteristic EEG patterns were identified that distinguish between types of depression with predominance of anxiety and inhibition.	EEG can help differentiate between types of depression in the elderly.
Fingelkurts et al. (2007)	Examine impaired functional connectivity in the alpha and theta EEG bands in major depression	Analysis of functional connectivity using alpha and theta waves of the traditional EEG	Communication disturbances in specific EEG bands can be a characteristic indicator of depression.
Cai et al. (2018)	Analysis of feature selection methods for the detection of depression using traditional EEG.	EEG data from three electrodes, different feature selection methods.	Beta wave activity, which is associated with mental activity and focus, may have atypical patterns in patients with depression.
Davidson (1998)	To discuss the model of anterior EEG asymmetries, affective style, and psychopathology, and to evaluate replication attempts of earlier findings.	Commentary based on two reports aiming to replicate previous results on EEG asymmetry, prefrontal and anterior temporal activation, and their relation to psychopathology.	Highlights the conceptualization of activation patterns across cortical-subcortical circuits. The causal role of EEG asymmetry in MDD
Li et al. (2017)	To examine changes in EEG phase synchronization during working memory processing in depressed patients.	Sixty-four-channel EEG was recorded from 33 depressed patients and 32 healthy controls during a visual n-back task.	Compared to controls, depressed patients showed reduced task-related increases in PSI for delta, theta, and alpha oscillations in the frontoparietal network, but elevated PSI for beta oscillations.
Lee et al. (2014)	Examine EEG patterns in depression co-occurring with internet addiction	Traditional EEG analysis in people with addiction and depression.	Studies have shown that patients with such a co-morbid diagnosis have an increased relative theta wave power and a reduced alpha wave power.
Kwon et al. (1996)	Evaluation of traditional EEG changes before and after treatment in patients with depression	Quantitative analysis of EEG before and after therapy.	Delta wave activity is increased in people with MDD
Spironelli et al. (2021)	Analyse delta and theta activity at rest in patients with MDD	EEG measurement at rest	Impaired delta and theta activity in patients
Saletu et al. (2010)	Evaluate traditional EEG and LORETA in patients with depression	60 female patients with depression + 30 controls	Increased relative values of delta/theta and beta waves, decreased alpha activity, slowing of the delta/theta centroid
Simmatis et al. (2023)	Review of technical and clinical aspects of EEG biomarkers in depression	Analysis of literature on traditional EEG as a biomarker of MDD	A greater delay in the delta wave phase has been observed in people with MDD
Buysse et al. (1997)	Investigate the relationship between EEG and susceptibility to recurrent depression	Analysis of sleep EEG in patients with depression	Lower delta activity as a potential marker for depression relapse
Nissen et al. (2001)	Assess whether NREM sleep characteristics predict the response to sleep deprivation (SD) in depression	Analysis of NREM sleep EEG before SD; comparison of patients responding and not responding to SD.	Sleep delta coefficient may be a predictor of the effectiveness of SD in the treatment of depression.
Jiang et al. (2021)	Develop a method for detecting depression using EEG and spatial information	Traditional EEG was used during the task with emotional faces	Classification with TCSP reached 84% (positive stimuli) and 85.7% (negative); the gamma band contributed the most;
Malik et al. (2018)	Early and precise detection of depression by measuring gamma wave activity in the brain	Analysis of gamma brain waves (20–30 Hz) using electroencephalography and the concept of a device that detects depression based on low gamma wave activity	Lower gamma activity may indicate depression; simple EEG device could be used for quick diagnosis
Pizzagalli et al. (2006)	Investigation of the relationship between depressive symptoms and ACC activity and error responses	128-channel resting state EEG + Flanker task; LORETA analysis	Lower gamma activity in the affective ACC may be responsible for difficulties in adapting after mistakes; potential indicator of response to depression treatment
Isomura et al. (2016)	Assess whether ASSR responses differentiate patients with MDD and BD, and compare them with a control group	306-channel MEG; analysis of ASSR and PLF responses to sound stimuli (20–80 Hz) in 14 patients with MDD, BD and 29 healthy subjects	Patients with MDD differed from BD in higher power and synchrony of ASSR in the 30–80 Hz band
Liu et al. (2012)	Investigate differences in local and far-reaching gamma oscillations in BD and MDD patients	MEG during an emotional task, time-frequency analysis of gamma responses	Patients with MDD show increased early gamma in the left anterior temporal region. Gamma patterns may differ
Lee et al. (2010)	Distinguish between brain activity in response to emotional stimuli in BD and MDD patients	Task with hidden facial emotions, time-frequency analysis and source imaging. 20 patients with BD, 20 with MDD, 20 healthy subjects	An increase in gamma in the bilateral temporal regions was observed in people with MDD
Mosabbir et al. (2022)	Evaluation of the effect of 5-week rhythmic vibroacoustic gamma stimulation (30–70 Hz) on depressive symptoms and changes in the traditional EEG	Auditory-tactile stimulation (RSS), resting EEG recording before and after intervention, assessment of changes in MADRS.	The Reduction of the Severity of Depressive Symptoms (MADRS) scale increased the occipital alpha and prefrontal gamma power, which suggests the effect of therapy on cortical activity.
Akdemir Akar et al. (2015)	Assessment of whether non-linear traditional EEG characteristics can differentiate between people with unipolar depression (MDD) and healthy people during emotional processing.	The study involved EEG recording during exposure to positive (music) and negative (noise) emotional stimuli. Patients with major depression (MDD) and healthy controls - number not specified	MDD patients show greater EEG signal complexity, especially in response to negative stimuli.
Korb et al. (2008)	Evaluate differences in cortical activity between people with MDD and healthy individuals.	Traditional EEG (36 channels) from 74 patients with MDD and a control group	MDD patients had a higher current density in the delta-beta2 bands in the anterior ACC and prefrontal cortex.
Roemer et al. (1992)	Assess QEEG in older people with depression	EEG measurement and topographic analysis	Lower delta values have been observed in patients

### 3.2 Asymmetry analysis

One of the frequently studied anomalies in MDD using EEG is asymmetry in brain activity, particularly in the alpha wave range. The researchers suggests that the relative difference between the left and right frontal brain regions in alpha wave activity may be a predictor of depression vulnerability. Withdrawal and negative behaviors are often related to high levels of right cortical activity, while positive reactions and mood have been related to increased left cortical activity. Therefore, frontal alpha asymmetry (FAA), with greater right-sided frontal activity, may be an important biomarker in diagnosing depression and anxiety (Barros et al., 2022). Alessandra Monni et al. analyzed a latent factorial approach to measure FAA. The research was conducted among 139 non-clinical participants. The authors distinguished a frontal alpha asymmetry factor (FAAf) and a parietal factor (PAAf) subjecting all asymmetry indices to a varimax-rotated, principal component analysis. Next the researchers explored among others the associations of latent factor and raw FAA scores with symptoms of depression and anxiety to determine which correlations were driven by FAA after variance from parietal activity was removed. Next, after correcting for false discovery rate, it turned out that only FAAf at the low alpha band was negatively correlated with depression symptoms (a latent CES-D factor) and significantly diverged from PAAf's association with depressive disorder. Therefore, the latent factor approach indicates beneficial effects for isolating functionally distinct resting-state EEG signatures (Monni et al., 2022).

The studies conducted by Ambrish Dharmadhikari et al. among 24 participants with Mild Depression indicates that results by measuring of FAA at resting stage was inconsistent. Researchers suggesting that it is necessary to revisit our approach from conventional search of diagnostic marker. Frontal Alpha Asymmetry might reflect component of depression but not the syndrome depressive disorder (Dharmadhikari et al., 2019). In turn, the meta-analysis by Luo et al., which includes 23 studies involving over 1,900 patients with MDD, showed that frontal alpha asymmetry measured with traditional EEG has a limited but statistically significant diagnostic value, which may support the clinical assessment of depression (Luo et al., 2025). It should be noted that frontal alpha asymmetry recorded during emotional stimulus processing differentiates individuals with MDD from healthy subjects more effectively than measurements taken at rest (Stewart et al., 2014; Périard et al., 2024). Arns et al. also investigated whether alpha wave asymmetry in the occipital and frontal regions of the brain could distinguish between outpatients with MDD and healthy individuals, assess the predictive power for antidepressant treatment outcomes, and account for the influence of gender on these relationships. The results did not show significant differences in alpha wave levels in the occipital and frontal cortex. However, for FAA, a specific interaction effect between gender and the type of medication used was observed. The findings suggest that future research on EEG alpha markers in depression should stratify participants by gender (Arns et al., 2016). Many researchers indicate a link between depression and EEG asymmetry, especially in the alpha and theta wave ranges in the frontal regions. In a study aimed at comparing the occurrence of regional brain asymmetries in severe depression with or without anxiety disorders, traditional EEG analysis of 44 patients revealed alpha asymmetry in individuals with MDD, indicating lower activation in the right posterior region compared to the left posterior region (Bruder et al., 1997). Another study showed that EEG spectral asymmetry (SA)—based on differences in power across frequency bands and their sources—differentiated women with depression from healthy individuals. This suggests that SA may serve as a useful marker of treatment outcome specifically in female patients (Hinrikus et al., 2010).

Abnormalities in traditional EEG activity are associated with various mental disorders, including depression, suicide and aggression. An EEG study conducted by Graae F. et al. showed differences in alpha wave asymmetry compared to the control group. Alpha asymmetry in the posterior regions of the brain was associated with suicidal ideation, but not with the severity of depression. The results suggest reduced activation of the left posterior brain as a factor associated with suicidal or aggressive behavior (Graae et al., 1996). Also, a reduction in the power of alpha wave activity in the left frontal lobe, which plays a key role in coping with emotional stress, may contribute to suicidal tendencies (Rasouli et al., 2024). This was also noted by Roh et al., who observed a reduced alpha wave power in the left frontal lobe in patients with MDD with suicidal thoughts compared to patients with MDD without such thoughts. Therefore, it can be seen that suicidal thoughts are an important moderator of alpha wave asymmetry in the frontal part of the brain in patients with MDD, as shown in Table 2 (Roh et al., 2020).

**Table 2:** Summary of EEG asymmetry analysis in patients with major depressive disorder.

**Researchers**	**Aim**	**Material and methods**	**Results**
Barros et al. (2022)	To compare frontal alpha asymmetry (FAA) in younger and older adults	Resting-state EEG analysis of 57 younger adults and 39 older adults. Regression analyses assessed the relationship between FAA and loneliness, depression, and anxiety.	Both groups showed greater left than right cortical activity. Older adults had higher FAA than younger adults.
Monni et al. (2022)	To assess frontal alpha asymmetry (FAA) using a latent factor approach, improving reliability and discriminant validity of resting-state EEG FAA measurements.	FAA was assessed at broad, low, and high alpha bands (8–13 Hz; 8–10.5 Hz; 11–13 Hz), using mastoid references and Current Source Density (CSD).	Both factor and raw scores showed excellent reliability, but only FAA demonstrated full discriminant validity. FAA at low alpha band was negatively associated with depression symptoms.
Dharmadhikari et al. (2019)	To evaluate the usefulness of frontal alpha asymmetry (FAA) as a potential biomarker for depression in both resting and activated EEG conditions.	24 patients with depression and 17 healthy controls were compared. EEG was recorded in resting, activated and post-activation phases. Alpha power at FP1, FP2, F3, F4, F7, and F8 was analyzed to assess FAA.	Significant FAA differences were found between groups at the F7–F8 pair and at F7 specifically. FAA varied across conditions, with the most pronounced differences during the activation phase.
Luo et al. (2025)	To assess frontal alpha asymmetry (FAA) as a potential resting-state diagnostic biomarker for major depressive disorder (MDD).	23 studies were included, with 1928 MDD patients and 2604 controls. FAA measurements were taken from EEG electrodes (F3/F4, F7/F8, or Fp1/Fp2).	FAA (F4 – F3) showed a small but significant overall effect size, suggesting limited diagnostic utility.
Stewart et al. (2014)	To test whether frontal EEG asymmetry during emotional challenge better reflects depression vulnerability than resting-state asymmetry.	EEG was recorded during rest and during a facial emotion task (approach emotions: anger, happiness; withdrawal emotions: fear, sadness). Asymmetry was analyzed using average, Cz, mastoid references	EEG asymmetry during the emotional challenge better distinguished MDD status than resting FAA for most references, supporting the capability model
Périard et al. (2024)	To investigate whether relative frontal alpha asymmetry (FAA) can serve as a biomarker for somatoform disorders (SFD) and its relationship with chronic stress and depressive symptoms.	Resting-state EEG was recorded using 64 electrodes (10-10 system) in 26 patients with primary SFD, 23 with major depressive disorder (MDD), and 25 healthy controls. FAA was calculated as alpha power in the right frontal cortex minus left.	No significant group differences in FAA were found. However, across all participants, lower relative left frontal activity was associated with higher chronic stress and depressive symptoms.
Arns et al. (2016)	Investigating EEG alpha asymmetry as a predictor of antidepressant treatment effectiveness.	EEG analysis in a randomized trial of treatment for depression (iSPOT-D)	Alpha asymmetry predicted treatment response
Bruder et al. (1997)	Investigate differences in EEG asymmetry between depressed patients with and without anxiety disorder.	Resting traditional EEG (eyes open and closed) in 44 patients with MDD (19 with anxiety, 25 without) and 26 healthy subjects	Depression with and without anxiety shows different brain activity patterns – in line with the model of hemispheric asymmetry
Hinrikus et al. (2010)	Study of EEG spectral characteristics in depression.	EEG spectral analysis in patients with depression.	EEG spectral characteristics showed significant differences between people with and without depression.
Graae et al. (1996)	Investigating alpha asymmetry in the traditional EEG of people who have attempted suicide	EEG analysis of people after a suicide attempt vs. a control group	Alpha asymmetry in the EEG may be a biomarker for suicide risk.
Rasouli et al. (2024)	Investigating EEG activity in the frontal regions during cognitive tasks in people who have recently attempted suicide.	Power spectrum analysis of EEG during Raven's task	Frontal EEG can be a useful indicator of suicide risk in people with depression.
Roh et al. (2020)	Investigating the role of frontal alpha asymmetry moderated by suicidal thoughts in MDD.	EEG analysis in MDD patients with and without suicidal thoughts	Frontal alpha asymmetry can help predict suicide risk in MDD

### 3.3 Functional connectivity analysis

Functional connectivity refers to the statistical relationships between EEG signals recorded from different brain regions, allowing for the assessment of the degree of their mutual synchronization and functional integration (Smailovic and Jelic, 2019).

Studies indicate that patients with depression may exhibit abnormalities in EEG signal coherence, particularly in high-frequency bands. In one experiment, Li et al. compared a group of healthy individuals and patients with MDD during an emotional face recognition task. The patient group showed significantly higher gamma-band coherence compared to healthy participants (Li et al., 2015). Similar results were observed in a study analyzing activity in the 35–45 Hz range among healthy individuals, those with depression, and those with schizophrenia—individuals with depression demonstrated increased gamma wave activity throughout the emotional task (Siegle et al., 2010). In addition to task-related activity, resting-state EEG also provides valuable diagnostic insights. In a study by Sun et al., the Phase Lag Index (PLI) was used to assess resting-state functional connectivity. The results showed that patients with depression had significantly disrupted intrahemispheric connectivity, particularly in the left hemisphere. The application of PLI achieved a classification accuracy of 82.3%, indicating its potential as a biomarker for MDD (Sun et al., 2020). Changes in connectivity are also observed during pharmacological treatment. Studies have shown that MDD patients who respond poorly to SSRI treatment exhibit stronger connectivity in the right frontotemporal network within the delta and theta bands (Lee et al., 2011). On the other hand, serotonin-norepinephrine reuptake inhibitors (SNRIs) tend to reduce theta-band coherence (Bares et al., 2008; Cook et al., 2002; Bares et al., 2015). In a review of studies, Armitage highlighted that individuals with depression show reduced interhemispheric coherence, particularly during sleep. The authors point out that computer-assisted EEG analysis can detect subtle changes in neural communication patterns that may underlie depressive symptoms and treatment outcomes, as shown in Table 3 (Armitage, 1995).

**Table 3:** Summary of functional connectivity analysis findings in major depressive disorder.

**Researchers**	**Aim**	**Material and methods**	**Results**
Li et al. (2015)	Evaluation of the structure of functional brain networks in patients with depression and healthy individuals during emotion processing using graph theory	Traditional EEG (59 electrodes); coherence and graph theory analysis (clustering, path length) in delta–gamma bands. 16 patients with depression, 14 healthy subjects	Depressed patients show higher coherence and more randomized network topology, especially in the gamma band
Siegle et al. (2010)	Investigate differences in emotional processing by analyzing gamma EEG after negative words in healthy people, people with depression and people with schizophrenia	Task: identifying emotions in words during traditional EEG; gamma band analysis (35–45 Hz). 24 healthy subjects, 14 patients with depression, 15 patients with schizophrenia	Depressed individuals showed prolonged and increased gamma activity after negative stimuli.
Sun et al. (2020)	To identify effective EEG biomarkers for recognizing depression, with a focus on functional brain connectivity features.	Resting-state EEG data were collected from 24 MDD patients and 29 healthy controls using a 128-channel HydroCel Geodesic Sensor Net.	PLI outperformed linear and nonlinear features. The highest classification accuracy (82.31%) was achieved using ReliefF feature selection and logistic regression.
Lee et al. (2011)	To assess whether the strength of functional EEG connections can predict the response to depression treatment after 8 weeks of SSRI treatment.	3-minute resting EEG (eyes closed) recorded in 108 patients with MDD. Connectivity strengths in responders and non-responders compared after 8 weeks	Stronger frontotemporal connections in the delta/theta band were associated with a poorer response to treatment
Bares et al. (2008)	To assess whether the decrease in QEEG theta coherence in the prefrontal brain area after 1 week of venlafaxine treatment can predict the clinical response in treatment-resistant patients.	25 hospitalized patients with MDD, QEEG recorded at baseline and after 1 week of treatment	An early decrease in the theta coherence may be a useful marker for predicting the effectiveness of venlafaxine
Cook et al. (2002)	To assess whether changes in QEEG theta-correlation in the prefrontal cortex can predict the clinical response to treatment with fluoxetine or venlafaxine.	51 patients with unipolar depression; EEG recorded at 3 time points	Only drug-responders showed a significant decrease in prefrontal coherence after 48 hours and 7 days
Bares et al. (2015)	To assess the effectiveness of QEEG theta-correlation in the prefrontal cortex as a predictor of response to venlafaxine ER in patients with MDD.	50 patients with MDD; QEEG performed at baseline, after 1 and 4 weeks	A decrease in coherence in the first week occurred in all responders in both groups
Armitage (1995)	Summary of 10 years of research on the microarchitecture of sleep in the traditional EEG of patients with depression	Review of sleep EEG studies in people with depression (both in episode and remission), compared with other clinical and control groups	Reduced delta activity in early sleep, increased fast EEG (especially in the right hemisphere) and reduced interhemispheric coherence are observed

### 3.4 Spatial analysis, machine learning

Source mapping of the brain's electrical activity responsible for the distribution of potentials on the scalp allows for the mathematical solution of the EEG inverse problem. One of the popular methods used is LORETA—a functional imaging technique that estimates the cortical sources of EEG signals in a three-dimensional brain model (Smailovic and Jelic, 2019). In depression, patients show reduced source current density in the left hemisphere. Differences in brain volume distribution are observed in the delta band, which is characterized by increased source current density, suggesting specific alterations in brain activity in this patient group (Flor-Henry et al., 2004). Bachmann and Lass identified EEG-based biomarkers of depression using a combination of two analysis methods: the linear Spectral Asymmetry Index (SASI) and the nonlinear Detrended Fluctuation Analysis (DFA). Results showed the most significant differences in SASI values in channel Pz and DFA values in channels Pz and O_2_, indicating that analyzing signals from a single parietal channel using these parameters may enable high classification accuracy between healthy individuals and those with depression (Bachmann et al., 2017). Additionally, patients with MDD exhibit higher Lempel-Ziv complexity (LZC) and lower power spectral density (PSD). The highest classification accuracy (up to 92.4%) was observed in the frontal, temporal, and central sources (Yang et al., 2023).

One study involving 36 participants analyzed brain activity changes before and after 12 weeks of antidepressant pharmacotherapy. It demonstrated that responders had lower multiscale entropy (MSE) at small scales and higher MSE at large scales, particularly in the fronto-central region (Jaworska et al., 2017). Korb et al. examined 74 individuals using 36-channel EEG and found significantly increased values in the delta, theta, alpha, beta1, and beta2 frequency bands in the anterior cingulate cortex and prefrontal cortex of patients with depression compared to the control group (Korb et al., 2008). In another study, Li et al. examined individuals with MDD using Event-Related Potential (ERP) measurements at frontotemporal sites. The results suggested that depressed participants had lower P300 amplitudes and significant differences in both fast and slow neural responses in the frontal and parietal lobes (Li et al., 2023). Bilateral projections of frontal activity and right parietotemporal activity were observed in participants with depression and comorbid disorders (Mathersul et al., 2008). It is worth noting that the use of simple EEG methods in combination with machine learning may serve as an effective diagnostic aid for depression. Ahmadlou et al. observed that individuals suffering from depression exhibit elevated gamma wave values in the frontal region. Their study also used Higuchi's fractal dimension (HFD) and Katz's fractal dimension (KFD) analyses (Ahmadlou et al., 2012). In another study, a simple 3-channel EEG system combined with linear and non-linear feature analysis and machine learning algorithms enabled discrimination between depressed and healthy individuals with a maximum classification accuracy of 76.4%, offering a potentially effective and accessible diagnostic tool for depression (Cai et al., 2018). In the study by Cai Hanshu et al., an EEG with three electrodes was used, placed at the points Fp1, Fp2 and Fpz. The collected data was analyzed using machine-learning based soft computing techniques. This approach enables automated QEEG signal denoising and categorization and in the cited study K-Nearest Neighbor (KNN), Support Vector Machine (SVM), Approximate Nearest Neighbor (ANN) and Deep Belief Network (DBN) classifying algorithms were used. The results showed that the DBN method outperformed traditional approaches using shallow algorithms. In addition, the researchers suggest that the absolute power of beta waves is one of the helpful indicators for detecting depression (Cai et al., 2016). Li et al. also noted after conducting an analysis that the average classification accuracy for the beta band was higher than for the alpha and theta bands in patients with depression which suggests that the beta band may be more suitable for machine-learning based detection of MDD features in QEEG than the other bands. However, this study was performed in individuals with mild form of depression, making its conclusion not necessarily applicable to MDD subjects (Li et al., 2019).

Traditional EEG studies conducted during depression treatment increasingly highlight characteristic changes in brain activity patterns influenced by different forms of pharmacotherapy. Analyzing these changes helps to better understand the neurophysiological mechanisms of antidepressant medications and identify potential markers of therapeutic efficacy (Leuchter et al., 2009a). Bruder et al. also observed that SSRI treatment resulted in increased alpha power among responders, particularly in the occipital region (Bruder et al., 2008). Detailed spatial EEG features and machine learning applications are presented in Table 4.

**Table 4:** Spatial EEG features and machine learning applications in the diagnosis of depression.

**Researchers**	**Aim**	**Material and methods**	**Results**
Flor-Henry et al. (2004)	Investigate the source of traditional EEG signals in men with depression	EEG tomography (LORETA) in drug-free patients	Hemispheric asymmetry has been observed, which may be characteristic of depression
Bachmann et al. (2017)	The objective of this study was to identify a simple and effective method for detecting depression based on the analysis of short, single-channel EEG signals.	The study involved 34 participants, including 17 diagnosed with depression and 17 healthy controls. EEG was recorded using 18 channels with a common Cz reference.	SASI values were significantly higher in the depression group compared to controls
Yang et al. (2023)	Evaluation of the influence of selected brain areas and combinations of regions on the effectiveness of MDD detection based on EEG	EEG analysis at rest (eyes closed/open)	Higher LZC and lower PSD in MDD; temporal region achieves 87.4% accuracy, frontal+temporal+central combination
Jaworska et al. (2017)	To assess whether the variability of the traditional EEG signal (MSE) at different time scales before antidepressant treatment can predict its effectiveness in people with MDD.	36 patients with MDD (untreated) and 36 healthy individuals. Resting EEG (eyes open/closed) was recorded before treatment.	Responders had lower MSE on small scales and higher MSE on large scales (especially frontocentral). These patterns did not occur in non-responders or the control group.
Korb et al. (2008)	Evaluate differences in cortical activity between people with MDD and healthy individuals.	Traditional EEG (36 channels) from 74 patients with MDD and a control group	MDD patients had a higher current density in the delta-beta2 bands in the anterior ACC and prefrontal cortex.
Li et al. (2023)	Investigate whether different levels of depressive states in healthy individuals are associated with different neuronal activity during the perception of emotional stimuli.	Healthy participants were divided into groups with low, medium and high levels of depression. ERPs and ERSPs were recorded during a visual perception task of emotional stimulation.	Individuals with high levels of depression showed a reduced P300 amplitude and differences in fast/slow neural responses in the frontal and parietal lobes.
Mathersul et al. (2008)	Investigation of the relationship between depression/anxiety and lateralisation of EEG activity in the frontal and parietotemporal regions.	Study on 428 people with varying levels of negative mood; EEG measurement (alpha waves) and lateralisation analysis were used.	In people with anxiety, right-sided frontal lateralisation was found, in depressive people - symmetrical frontal activity and increased right parietal-temporal activity.
Ahmadlou et al. (2012)	Investigation of the complexity of frontal EEG signals in MDD patients using non-linear methods (HFD, KFD)	Traditional EEG divided into 5 sub-bands of brainwave frequencies; KFD and HFD were calculated, statistically compared (ANOVA), and then used in the EPNN classifier	HFD revealed greater complexity in the frontal regions of the brain of MDD patients, especially in the beta and gamma bands. HFD beta differentiated MDD from healthy subjects particularly well.
Cai et al. (2016)	To enhance the accuracy of detecting mild depression using EEG by applying differential evolution for feature optimization and k-nearest neighbors for classification.	EEG data from 10 individuals with mild depression and 10 healthy controls were analyzed. Differential evolution was used to optimize the extracted EEG features	Combining differential evolution with k-NN classification enhances the detection of mild depression from EEG data.
Li et al. (2019)	To develop an accurate and portable diagnostic method for depression using a three-electrode EEG setup and compare the performance of various classification algorithms.	EEG data were collected from 178 participants using three scalp electrodes placed at Fp1, Fp2, and Fpz—regions closely related to emotion and unobstructed by hair. The algorithms used for classification included k-NN, SVM, ANN, and DBN	The Deep Belief Network (DBN) achieved the highest accuracy (78.24%) when combined with absolute beta wave power.
Leuchter et al. (2009a)	Assessment of the usefulness of the ATR index from QEEG in predicting response to various antidepressants in patients with MDD	375 patients with MDD; QEEG before and after a week of escitalopram (10 mg), then randomized to: escitalopram, bupropion or a combination of the two	High ATR predicted the effectiveness of escitalopram (68% vs. 28%); low ATR – greater effectiveness of bupropion after changing treatment (53% vs. 28%)
Bruder et al. (2008)	To investigate whether the resting power and asymmetry of EEG alpha waves differ between depressed patients who respond and do not respond to SSRI treatment and whether it changes after treatment.	18 patients with depression and 18 matched healthy individuals	Responders had greater alpha power, especially in the occipital regions. Alpha asymmetry (greater power on the right) was observed in responders, unlike in non-responders

## 4 Discussion

### 4.1 Summary of main findings

Depressive disorders are among the most commonly diagnosed mental disorders worldwide, but they remain difficult to diagnose accurately, especially in cases with a mild or atypical course. Current traditional diagnostic methods, which rely on clinical interviews and rating scales, are prone to errors due to subjective opinion. Therefore, they do not provide a complete picture of the neurobiological basis of depression. For this reason, increasing attention is now being paid to objective methods which include the analysis of bioelectrical brain activity by means of EEG and QEEG (Kopańska et al., 2023; Williams, 1954).

This article analyses the results of a study that looked for patterns of brain wave activity characteristic of patients with major depression. The analysis focuses on specific EEG bands which include alpha, beta, theta, delta and gamma waves. The most common phenomenon observed by other researchers was the asymmetry of alpha waves in the prefrontal regions (with dominant activity on the right side), which seems to be related to the severity of depressive symptoms and the presence of suicidal thoughts (Graae et al., 1996; Rasouli et al., 2024; Roh et al., 2020). In the case of waves, their increased activity has been shown in some studies to be a positive predictor of response to treatment, especially drug treatment (Grin-Yatsenko et al., 2009; Suzuki et al., 1996). In contrast, gamma waves, which are associated with the integration of cognitive and emotional information, tend to decrease in patients with MDD (Jiang et al., 2021; Malik et al., 2018; Pizzagalli et al., 2006; Akdemir Akar et al., 2015).

It is worth noting that the results of traditional EEG studies in depression are not entirely consistent (Arns et al., 2015). An additional complication is that many studies rely on small samples and different analytical techniques (including band power analysis, coherence, source activity mapping and non-linear indices). Despite these limitations, EEG/QEEG has not only diagnostic but also prognostic potential. Changes in specific wavebands prior to treatment can serve as predictors of treatment efficacy—for example, higher theta activity and lower gamma activity prior to SSRI treatment were associated with better response to pharmacotherapy (Leuchter et al., 2009a). Schiller noted that the use of QEEG in depressed patients may help to adjust pharmacotherapy, especially in treatment-resistant cases (Schiller, 2019). Additionally, the increasing importance of combining QEEG with advanced computational tools, such as machine learning algorithms or signal source localization methods, deserves special attention. These approaches not only improve the accuracy of classification between MDD patients and controls, but also open new perspectives for medicine in psychiatry (Cai et al., 2018; Ahmadlou et al., 2012). In conclusion, quantitative electroencephalography is a promising tool to aid the diagnosis of depression.

## 5 Restrictions

Despite the interesting results obtained, this study has certain limitations that should be taken into account when interpreting the data. First, the source studies analyzed in this review differed significantly in terms of methodology, making it difficult to directly compare the results. Variability concerned both the study populations (age, gender, severity of depressive symptoms, presence of comorbidities) and the technical parameters of QEEG number and placement of electrodes, recording conditions, signal length. Secondly, some of the studies analyzed were based on small groups of participants, which limits the possibility of generalizing the results to the general population. It is also worth noting that not all studies took into account the control of significant confounding variables, such as the use of psychotropic drugs, which can affect EEG activity. In addition, it is worth noting that most studies are cross-sectional, which limits the possibility of drawing causal conclusions. Further studies with a large number of participants and standardized protocols for quantitative electroencephalography recording are needed to better understand the role of bioelectrical brain activity patterns in depression.

## 6 Conclusion

Based on the studies and literature analyzed in this study, it appears that the treatment of depression requires an interdisciplinary approach combining pharmacotherapy, psychotherapy and advanced diagnostic techniques. Therefore, research on biomarkers of depression can open new perspectives for personalized medicine, offering the possibility of tailoring therapy to the individual needs of the patient. After analyzing the available publications, it can be seen that quantitative electroencephalography and traditional EEG examination are promising tools in the diagnosis and monitoring of depression. The data collected in this study indicate that the analysis of bioelectric brain activity can provide valuable information on specific neurophysiological changes associated with depression. The results of the study suggest that abnormalities in brainwave frequency bands – especially alpha wave asymmetry, increased beta activity and changes in the theta and delta waves – may be potential biomarkers for depression. Some studies have shown that selected EEG and QEEG parameters can distinguish individuals with depression from healthy controls with high accuracy—for example, the use of the Phase Lag Index (PLI) achieved a classification accuracy of over 80%. Despite these promising results, there is still a lack of standardized procedures and analysis protocols, which limits the practical application of these methods in clinical diagnostics.
